# Diagnostic and Therapeutic Implications of Sortilin Expressed on the Surface of Bladder Carcinoma Cells

**DOI:** 10.30699/IJP.2022.539411.2732

**Published:** 2022-03-08

**Authors:** Ali-Ahmad Bayat, Niloufar Sadeghi, Ghazaleh Fazli, Mohammad Reza Nowroozi, Solmaz Ohadian Moghadam, Amin Radmanesh, Mohammadjavad Hedayatshodeh, Ali Reza Sarrafzadeh, Omid Zarei, Fatemeh Ghaemimanesh, Hodjattallah Rabbani

**Affiliations:** 1 Monoclonal Antibody Research Center, Avicenna Research Institute, ACECR, Tehran, Iran; 2 Uro-Oncology Research Center, Tehran University of Medical Sciences, Tehran, Iran; 3 Legal Medicine Research Center, Legal Medicine Organization, Tehran, Iran; 4 Department of Tissue Engineering and Applied cell sciences, Shahid Beheshti University of Medical Sciences, Tehran, Iran; 5 Department of Pathology, Khatam Al Anbia Hospital, Tehran, Iran; 6 Cellular and Molecular Research Center, Research Institute for Health Development, Kurdistan University of Medical Sciences, Sanandaj, Iran

**Keywords:** Bladder cancer, Flow cytometry, Monoclonal antibody, Sortilin

## Abstract

**Background & Objective:**

Cell surface expression of sortilin in different types of cancer signifies it as a therapeutic target for cancer therapy. The aim of this study was to detect sortilin expression in bladder cancer cells using an anti-sortilin monoclonal antibody (mAb) to evaluate sortilin as a target for developing diagnostic and therapeutic agents against bladder carcinoma.

**Methods:**

The protein expression of sortilin in bladder cancer tissues and cell lines (5637 and EJ138) was investigated by immunohistochemistry (IHC), immune-cytochemistry (ICC), and flow cytometry. Furthermore, the capability of anti-sortilin mAb in apoptosis induction in bladder cancer cells was evaluated.

**Results:**

A high expression level was observed in bladder carcinoma tissues (*P*≤0.001) and cell lines, using IHC and ICC, respectively. Flow cytometry results showed cell surface expression of 27.5±3% (*P*≤0.01), 74.4±7.8% (*P*≤0.001), and 4.2±0.4% of sortilin in EJ138, 5637, and HFFF cells, respectively. In EJ138 anti-sortilin mAb induced apoptosis in 25.2±11.5% (*P*≤0.05) (early) and 4.5±1.1% (*P*>0.05) (late) after 6 h incubation, while for 12 h, the values of 11.6±3.8% (*P*>0.05) and 20.7±4.4% (*P*≤0.05) were achieved. In 5637 cells, 6 h incubation resulted in 10.2±0.3% (*P*>0.05) and 6.6±1.4% (*P*>0.05) apoptosis induction, while these values were 12.1±0.8% (*P*>0.05) and 27.4±4.5% (*P*≤0.01) after 12 h. The HFFF cells did not show significant apoptosis.

**Conclusion:**

The overexpression of sortilin in bladder tumor cells and its potential in inducing apoptosis via directed targeting with the specific monoclonal antibody may represent this protein as a potential candidate of targeted therapy in bladder carcinoma.

## Introduction

Cancer is the second cause of human death world-wide, with more than 9.5 million cases per year. Among those, about 200.000 cases are bladder cancers. Bladder cancer is the fourth most common malignant with most male patients (1). This malignancy is classified into muscle-invasive and non-muscle invasive, while the first one is linked to death through metastases, and the second one is known for tending to recurrence (2). Histologically, urothelial carcinoma or transitional cell carcinoma constitutes more than 90% of bladder cancer cases, while squamous and adenocarcinomas of the bladder constitute the rest (3). For treatment of bladder cancer, chemotherapeutic agents and immunotherapy interventions such as nivolumab, pembrolizumab, atezoli-zumab, durvalumab, and avelumab are available (4). Among cancer therapy strategies, the passive immune-therapies by monoclonal antibodies targeting cell surface antigens gained attention to combat different types of malignancies (5-7). Numerous studies have demon-strated therapeutic and prognostic values of biomarkers in urothelial carcinoma and other urinary tract tumors, like PMSA in prostate cancer (8) CXR2 and CXR3 in renal cell carcinoma (9) and CD38 and Zap-70 in CLL (10). Consequently, sortilin and its functional role in bladder cancer might be considered as a novel diagnostic and therapeutic agent in bladder carcinoma.

Sortilin, also known as Neurotensin Receptor-3 (NTR3), is a multi-ligand receptor and a member of the Vacuolar Protein Sorting 10 (VPS10) family of sorting receptors that is involved in various biological processes (11-15). Sortilin is encoded by the *SORT1* gene located on the short arm of chromosome 1 (1p13-3). Structur-ally, sortilin is a type I transmembrane glycoprotein receptor with an extracellular domain, a single transm-embrane helix, and a short cytoplasmic tail (16, 17). 

The overexpression and dysregulation of sortilin in several human malignancies have been reported previo-usly, representing sortilin as a cell surface protein appro-priate for targeted immunotherapy using a monoclonal antibody (18-20). Although the expression of sortilin in different types of cancer has been reported (18, 21, 22), there is no study regarding its expression in bladder carc-inoma. This notion encouraged us to study the sortilin expression in bladder cancer cells and primary tumor tissues by IHC, ICC, and flow cytometry techniques to find a novel diagnostic method and a novel target to combat this malignancy.

## Material and Methods

In our previous study, we produced a monoclonal antibody called 2D8–E3 against a synthetic peptide derived from the first 50 amino acids of the extrace-llular domain of sortilin, capable of recognizing its corresponding protein (23). This study was performed to develop a detection method as well as evaluate a possible immunotherapeutic target in bladder carcin-oma at Avicenna Research Institute.

**Cell Culture**

RPMI-1640 medium, fetal bovine serum (FBS), penicillin, and streptomycin were purchased from Gibco, NY, USA. Human bladder carcinoma cells lines EJ138 (NCBI Code: C429; ECACC Number: 850611-08), 5637 (NCBI Code: C450; ECACC Number: DSMZ NO: ACC 35), and human Caucasian fetal foreskin fibroblast (HFFF, NCBI Code: C107) cells were obtained from National Cell Bank of Iran (Pasteur Institute, Tehran, Iran). All cell lines were cultured in RPMI-1640 containing 10% FBS, penicillin (100 U/mL), streptomycin (100 µg/mL) and incubated at 37°C with 5% CO_2_ and 95% humidity (24).

**Immunohistochemistry (IHC)**

Formalin-fixed paraffin-embedded (FFPE) from human bladder carcinoma (n=23) and normal bladder tissue samples (n=20) were received from Imam Khomeini hospital, Tehran, Iran, and National Forensic Organization, Tehran, Iran, respectively. Tissues were deparaffinized and prepared for immunostaining accor-ding to our previous report (25). To quench the endo-genous peroxidase activity, a 3% H_2_O_2_ was used. To block the nonspecific binding sites, a 5% normal sheep serum in Tris-buffered saline containing 2.5% Bovine serum albumin (2.5% TBS-BSA) was used. The slides were then incubated with 10 µg/mL anti-sortilin mAb (PadzaCo., Tehran, Iran) in a 2.5% TBS-BSA or anti-beta actin or mouse IgG isotype control antibodies (PadzaCo., Tehran, Iran) at room temperature (RT) for 60 min. The slides were washed by 0.1% TBS-BSA for three times, and 50 µL of EnVision reagent (BioGenex, United States) was added to the slides with an incubation time of 30 min at RT. Then, 50 µL of DAB substrate (3, 3'-diaminobenzidine) (BioGenex, United States) (1:50 dilution, according to the manufacturer instructions) was added to each slide with subsequent adding hematoxylin dye (Merck, Darmstadt, Germ-any). Finally, the slides were washed with deionized water, followed by dehydrating with ethanol, mounting by Entellan (Merck, Darmstadt, Germany). The slides were examined using fluorescent microscopy (Olympus, Tokyo, Japan). 

**Immunocytochemistry (ICC)**

The cells were seeded at a concentration of 2×10^4^ cells/well in 100 µL medium on an eight wells glass coverslip (Germany, Marienfeld GmbH, Lauda-König-shofen) and incubated overnight with complete RPMI-1640 (supplemented with FBS, penicillin, and strepto-mycin) at 37°C in 5% CO_2_ with humidity atmosphere. Then cells were fixed by acetone for 10 min, blocked with 5% normal sheep serum for 30 min, and incubated with 5 µg/mL sortilin mAb as well as IgG isotype control for 45 min at RT. The slides were further incubated with secondary FITC-conjugated sheep anti-mouse Ig (PadzaCo., Tehran, Iran) at a dilution of 1:50 for an additional 30 min. The nuclei were stained with 1 µg/mL 4`,6-diamidino-2-Phenylindole (DAPI) (USA, Calbiochem) stain for 5 min. The slides were examined under a fluorescent microscope (*Olympus BX51*, Tokyo, Japan) (26).

**Flow Cytometry**

All three cell lines were cultured to reach 70–80% confluency. The cells were harvested and washed using pre-cold phosphate-buffered saline (PBS) and blocked with 5% sheep serum at 4°C for 30 min. The cells were then treated for 1 h at 4°C by 10 µg/mL concentration of anti-sortilin mAb or equivalent concentration of IgG isotype control antibody. The cells were washed as described above, incubated with FITC-conjugated sheep anti-mouse for 45 min at 4°C (in a dark place), at 1:50 dilution. The washing was repeated, and the fluorescence was measured using a Partec PAS® flow cytometer (Partec® GmbH, Münster, Germany). The results were analyzed with FloMax software (Partec, Nuremberg, Germany). To determine the relative total cell surface expression of sortilin, the mean fluore-scence intensity (MFI) was multiplied by the perce-ntage of positivity (POP) (MFI×POP) (27). 

**Apoptosis Assay**

A total number of 1.5x10^5^ cells from each cell line were cultured in a 24 well plate in RPMI- 1640 medium. The procedure was followed by treatment with 10 µg/mL of 2D8-E3 mAb or an isotype control IgG for 6 and 12 h in a serum-free medium. The cells were harvested and washed twice with cold PBS. Finally, 100 µL of the cell suspension in 1x binding buffer was stained with 1 µL Annexin V- FITC (BD Biosciences, San Jose, CA) and 2 µL propidium iodide (PI) (BD Biosciences, San Jose, CA), followed by a short vortex and incubation at RT for 15 min in a dark place. The percentage of early apoptotic cells (Annexin V^+^, PI^-^) and late apoptotic cells (Annexin V^+^, PI^+^), as well as live cells (Annexin V-, PI-), were observed using Partec PAS III flow cytometer (Partec GmbH, Germany) and analyzed by FlowJo software (version 10) (28). 

**Statistical Analysis**

The statistical analysis was carried out by one-way and two-way ANOVA using GraphPad Prism software. The results were illustrated as mean ± SD. The P-values less than 0.05 were considered statistically significant**.**

## Results

**Immunohistochemical Staining for Evaluation of Sortilin Expression**

**Fig. 1:**
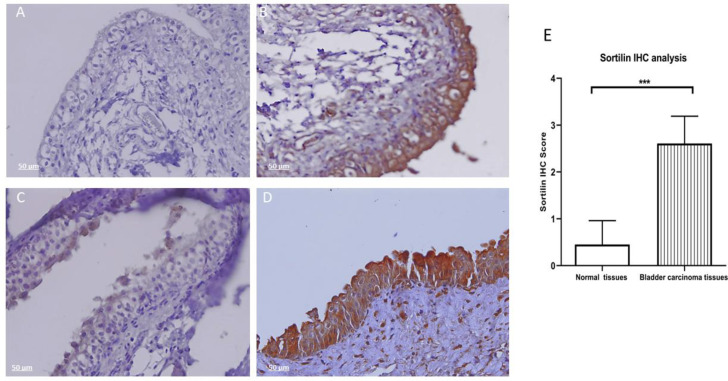
Sortilin expression by immunohistochemistry (IHC). Formalin-fixed paraffin-embedded (FFPE) human bladder carcinoma and normal bladder tissues were incubated with anti-sortilin mouse monoclonal antibody (2D8-E3), mouse IgG isotype, and anti-beta actin antibodies. The signals were detected using EnVision system (BioGenex, United States). Counterstaining was performed by Mayer's hematoxylin. A) IgG Isotype control antibody and bladder carcinoma tissue. B) Anti-beta actin antibody and bladder carcinoma tissue. C) Anti-sortilin antibody and normal bladder tissue. D) anti-sortilin antibody and bladder carcinoma tissue (Original magnification, ×50). E) Sortilin IHC analysis revealed that the expression of sortilin in the transitional epithelium in all human bladder carcinoma tissues (n=23) is significantly higher than normal bladder tissues (n=20) (***: *P*≤0.001). Scoring was based on the intensity of DAB staining (rating from 0-4 plus). Normal samples (0-1+) and positive cases (≥2+)

IHC results revealed that the expression of sortilin in the transitional epithelium in all human bladder carcinoma tissues is significantly higher than in normal bladder tissues (*P*≤0.001) (Figure 1). 

**Detection of Sortilin by Immunocytochemistry**

**Fig. 2:**
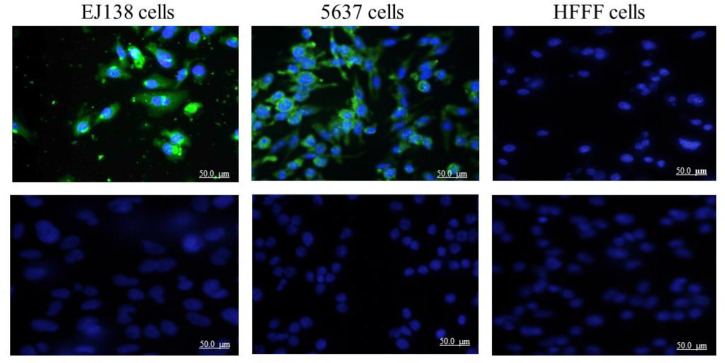
Immunocytochemistry staining of sortilin in EJ138, 5637, and HFFF cells (A-C) using anti-sortilin monoclonal antibody clone 2D8-E3 as primary and FITC-conjugated sheep anti-mouse antibody as secondary antibodies as well as DAPI (for the nucleus staining). Green fluorescence represents sortilin expression, and blue color indicates the nucleus. IgG isotype control was used instead of anti-sortilin antibody (D-F) (Original magnification, ×50)

The expression of sortilin in bladder cancer cells was also investigated by immunofluorescent staining using anti-sortilin mAb (2D8-E3). Both EJ138 and 5637 cells showed expression of sortilin (the green color) detected by their interaction with 2D8-E3, while no signal was detected in the normal cell line (HFFF) (Figure 2). 

**Cell Surface Sortilin Detection by Flow Cytometry**

**Fig. 3:**
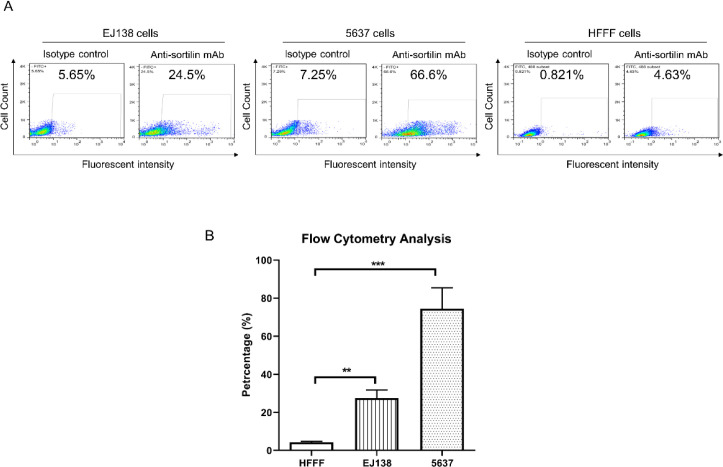
Flow cytometry analysis of cell surface sortilin expression in bladder cancer cell lines using anti-sortilin monoclonal antibody clone 2D8-E3. The values for isotype control are also demonstrated. In part A, cell surface expression of sortilin was observed in EJ138, 5637, and HFFF cells. In part B, The bar graph of sortilin expression average in 5637, EJ138, and HFFF cells (**: *P*≤0.01; ***: *P*≤0.001)

Flow cytometry assay using 2D8–E3 mAb was performed to detect the cell surface expression of sortilin in bladder cancer cell lines. Results revealed that sortilin is significantly overexpressed on the surface of EJ138 (count: 27.5±3%, *P*≤0.01) and 5637 (count: 74.4±7.8%, *P*≤0.001) bladder carcinoma cell lines. In comparison, HFFF normal control cell line expressed low amount of sortilin (count: 4.2±0.4%). The arbitrary values of MFI×POP for EJ138, 5637, and HFFF cells were 610.5, 2641.2, and 10.24, respectively (Figure 3 and Table 1).

**Table 1:** Flow cytometry on the bladder cancer and normal cell lines

Cell line	Antibody	MFI	Mean of POP±SD	MFI×POP	P-value
EJ138	Anti-sortilin mAb	22.2	27.5±3%	610.5	**P≤0.01**
5637	Anti-sortilin mAb	35.5	74.4±0.7%	2641.2	**P≤0.001**
HFFF	**Anti-sortilin mAb**	**2.44**	**4.2±0.4%**	**10.24**	**-**

mAb = Monoclonal antibody POP= Percentage of positivity

MFI = Mean fluorescence intensity SD= Standard deviation

**Apoptosis Detection by Flow Cytometry**

**Fig. 4:**
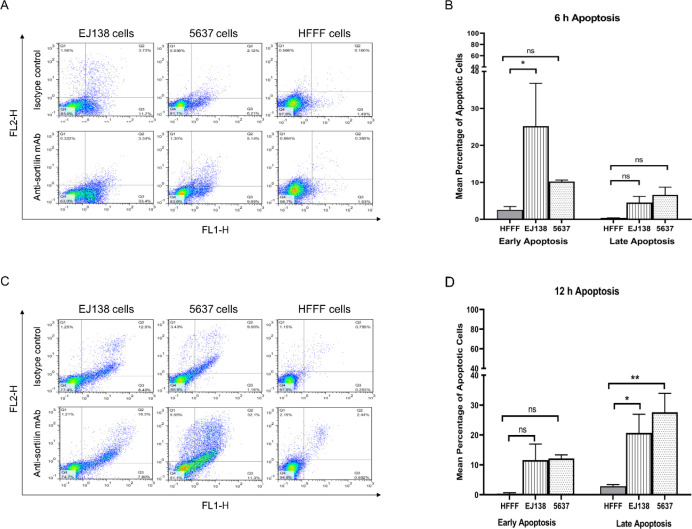
Apoptosis induction in two bladder cancer cell lines (EJ138 and 6537) after 6 and 12 h treatment by anti-sortilin monoclonal antibody clone 2D8-E3. The percentage of viable cells for EJ138 and 5637 cells were 63% and 83.6% after 6 h and 74.7% and 51.1% for 12 h treatments. In part A, the rate of early and late apoptosis in both cells as well as the obtained values for isotype control mAb for 6 h incubation, are illustrated. B, the bar graph of apoptosis induction for 6 h incubation by anti- sortilin mAb. C, the same experiment after 12 h treatment, the percentage of early and late apoptosis in both cells and isotype control mAb are demonstrated. D, the bar graph of apoptosis induction for 12 h incubation by anti- sortilin mAb. (*: *P*≤0.05; **: *P*≤0. 01, ns (not significant) = *P*>0.05)

EJ138, 5637, and HFFF cells were treated with anti-sortilin mAb for 6 and 12 h. The results indicated apoptosis induction in both carcinoma cell lines. After 6 h incubation, 25.2±11.5% (*P*≤0.05) (early) and 4.5±1.1% (*P*>0.05) (late) apoptosis was detected, while for 12 h incubation, the values of 11.6±3.8% (*P*>0.05) (early) and 20.7±4.4% (*P*≤0.05) (late) were achieved in EJ138 (Figure 4). In the case of 5637 cell line, 6 h incubation resulted in 10.2±0.3% (*P*>0.05) (early) and 6.6±1.4% (*P*>0.05) (late) apoptosis induction, while these values were 12.1±0.8% (*P*>0.05) (early) and 27.4±4.5% (*P*≤0.01) (late) after 12 h incubation. The HFFF cells almost did not show significant apoptosis. Only 2.1±1.5% (early) and 0.2±0.1% (late) of apoptotic cells were detected following 6 h incubation. These values were 0.3±0.2% (early) and 2.8±0.3% (late) after 12 h incubation.

## Discussion

The present study showed that sortilin is overe-xpressed in both tissues and cell lines of bladder carcinoma using three read-out systems, including IHC, ICC, and flow cytometry by anti-sortilin mAb. Although several other therapeutic and prognostic biomarkers cancers have been documented in bladder cancer (29), it is for the first time that the overexpression of sortilin is reported in bladder carcinoma. Sortilin is part of the machinery that serves essential and diverse functions in pathologic conditions. The IHC results obtained from analysis of all bladder tissue samples indicate that the overexpression of sortilin is highly restricted to the transitional epithelium of bladder carcinoma with no expression in the underlying connective tissues. A similar pattern with high expression of sortilin in the epithelial layer but not in its underlying connective tissue was reported by Boggild, demonstrating sortilin overexpression in epithelial tissues including pancreas, kidney, developing lung, nasal cavity salivary gland, and intrahepatic bile ducts. This finding may imply that sortilin could participate in the trafficking and regulation of different proteins involved in organ development (30). 

Our flow cytometry results showed that the expression of sortilin on the surface of both bladder carcinoma cell lines (5637 and EJ138) is more than the normal fetal foreskin fibroblast cell line (HFFF);. However, the expression in the 5637 cell line is higher than EJ138. This finding may be due to the chara-cteristics of each cell line representing its particular phenotype and duty. Phenotypically, EJ138 is a transitional bladder cell carcinoma and known as an invasive cell line, while the 5637 is a grade II bladder carcinoma and known to be a non-invasive line (31, 32). Furthermore, the findings indicated higher cell surface expression of sortilin in cancer cell lines than normal counterparts, which may be due to elevated translocation of sortilin to the surface of cancer cells. Previously, it has been reported that only 10% of total sortilin could translocate to the surface of normal cells while 90% remained in cytoplasmic organelles such as trans-Golgi-network and vesicles (33, 34). Such significant variation in cell surface expression of sortilin between normal and cancer cells makes sortilin an ideal target for cancer diagnosis and therapy. Significant expression of VEGF has been reported to be associated with low-grade bladder carcinoma (9), which would be a good candidate to be used along with sortilin as a prognostic and therapeutic marker. So, concurrent use of anti-sortilin and anti-VEGF anti-bodies for targeted immunotherapy of bladder carcin-oma is suggested. The overexpression of sortilin and its clinicopathological role in cancer has been reported in different types of human solid cancers, including breast (18, 35, 36), neuroendocrine (20), ovarian (25, 37), colorectal (38), and hematological malignancies such as chronic lymphocytic leukemia (CLL) (39) during the last decades. 

In this study, we also evaluated the functional role of sortilin in the pathophysiology of bladder carcin-oma, using apoptosis induction assay with anti-sortilin mAb in cell culture. The results showed that anti-sortilin mAb induced apoptosis in both EJ138 and 5637 cell lines after 6 and 12 h treatment without significant effect on human normal HFFF cells. Interestingly, the early apoptosis rate was similar in both cells after 6 h, while the percentage of late apoptosis was higher in 5637 cells than EJ138 cells. After 12 h, the early and late apoptosis rate was elevated in 5637 cells compared to EJ138 cells. However, no apoptosis was detected in comparison with an isotype control. The observation of apoptosis in EJ138 after 6 h might reflect the role of sortilin as a survival factor in bladder cancer cells. The notion has previously been described for other cancer cell types like neuroendocrine tumors and B cell malignancies (20, 40). Several studies suggested that antibodies can induce apoptosis via blocking the ligand-receptor growth, survival pathways, antigen crosslinking, the activation of death receptors, or hyperpolarization of mitochondrial membranes in cancer cells (41, 42). Although the exact mechanism by which anti-sortilin mAb induces apoptosis in bladder carcinoma has not yet been elucidated, the vital role of sortilin in the pathology of bladder cancer is currently evident by achieved data from this study. The mechanisms by which sortilin facilitates oncogenesis and cancer progression are completely different in various cancer cells. For instance, the function of sortilin in transport of neurotensin as a peptide that induces tumor growth and proliferation has been well documented in pancreas, colon and prostate cancers (43, 44). A similar study showed that the internali-zation of neurotensin by sortilin induced migration of human microglial cells through the stimulation of both MAP and Pi3-kinase-dependent pathways (45). Other studies demonstrated that cooperation between sortilin and TrkA facilitates the invasion of breast carcinoma by activating Akt and Src molecules via binding with the cancer-accessory factor of proNGF (35, 46). 

Some reports demonstrated that sortilin is a contributory factor in expanding cancer stem cells. This is due to the role of sortilin in the internalization of progranulin as a glycoprotein involved in the transforming machinery and propagation of cancer stem cells (36, 47). In breast cancer, progranulin associated with sortilin induces propagation of cancer stem cells and dedifferentiation of well-differentiated cells (36). Therefore, the authors suggested that prevention of sortilin might inhibit its communication with progranulin, and potentially it might be used as a bladder cancer therapeutic strategy. Altogether, it seems that sortilin is playing a mediator role in different cancer progression pathways. In case of overexpression of tumor markers such as sortilin with low expression in normal cells, a conjugation of antibody with chemical drugs (antibody-drug conju-gates, ADC) in the format of either mono- or multi-targeting is proposed.

## Conclusion

The overexpression of sortilin in bladder tumor cells and its potential in inducing apoptosis via directed targeting with the specific monoclonal antibody may represent potent as a potential candidate of targeted therapy in bladder carcinoma.

## Conflict of Interest

The authors declare no conflict of interest.

## Funding

None.
